# Pleural fluid analysis of lung cancer *vs* benign inflammatory disease patients

**DOI:** 10.1038/sj.bjc.6605607

**Published:** 2010-03-09

**Authors:** R Kremer, L A Best, D Savulescu, M Gavish, R M Nagler

**Affiliations:** 1 Department of General Thoracic Surgery, Rambam Health Care Campus, Haifa, Israel; 2 Oral Biochemistry Laboratory, Rappaport Faculty of Medicine, Molecular Pharmacology Department, Technion – Israel Institute of Technology Haifa, Haifa, Israel; 3 Oral and Maxillofacial Surgical Department, Rambam Medical Center, Haifa, Israel

**Keywords:** pleural fluid, lung cancer, inflammation, pleural effusion, cytology, tumour markers

## Abstract

**Background:**

Correct diagnosis of pleural effusion (PE) as either benign or malignant is crucial, although conventional cytological evaluation is of limited diagnostic accuracy, with relatively low sensitivity rates.

**Methods:**

We identified biological markers accurately detected in a simple PE examination. We analysed data from 19 patients diagnosed with lung cancer (nine adeno-Ca, five non-small-cell Ca (not specified), four squamous-cell Ca, one large-cell Ca) and 22 patients with benign inflammatory pathologies: secondary to trauma, pneumonia or TB.

**Results:**

Pleural effusion concentrations of seven analysed biological markers were significantly lower in lung cancer patients than in benign inflammatory patients, especially in matrix metalloproteinase (MMP)-9, MMP-3 and CycD1 (lower by 65% (*P*<0.000003), 40% (*P*<0.0007) and 34% (*P*<0.0001), respectively), and in Ki67, ImAnOx, carbonyls and p27. High rates of sensitivity and specificity values were found for MMP-9, MMP-3 and CycD1: 80 and 100% 87 and 73% and 87 and 82%, respectively.

**Conclusion:**

Although our results are of significant merit in both the clinical and pathogenetic aspects of lung cancer, further research aimed at defining the best combination for marker analysis is warranted. The relative simplicity in analysing these markers in any routine hospital laboratory may result in its acceptance as a new diagnostic tool.

Paramount importance lies in the correct diagnosis of pleural effusion (PE) as benign or malignant, as PE might be the first presenting sign of cancer, suggestive of recurrent or advanced disease (DeCamp *et al*, 1997), naturally necessitating adequate treatment and management. Malignant pleural effusion (MPE) is a common and distressing condition seen at the advanced stage of various malignant diseases, with the majority of cases having exsudative character (Valdés *et al*, 1996). Although lung cancer is the most common cause of MPE (Neragi-Miandoab, 2006), almost all forms of cancer, including cancer of ovary and stomach, lymphoma, Hodgkin's and non-Hodgkin's disease, can cause MPE as well (Sahn, 1998). As for the formation of MPE, primary or metastatic tumours may invade the visceral pleura, affecting the normal resorptive flow of fluid from the parietal to the visceral pleura (DeCamp *et al*, 1997) or causing increased capillary leaking and increased fluid production (Silverberg, 1970). A blockage in the lymphatic system anywhere between the parietal pleura and mediastinal lymph nodes results in accumulation of fluid in the pleural space (Meyer *et al*, 2004). Intrapulmonary shunt is the main underlying reason for the arterial hypoxaemia associated with large PE (Perpiñá *et al*, 1983). Benign PE can possibly result from inflammatory processes, as well as from left ventricular failure, cirrhosis and haemodynamic or neoplastic conditions (Woenckhaus *et al*, 2005).

The conventional cytological evaluation of pleural fluids for differentiating benign PE from MPE is of limited diagnostic accuracy (García-Bonafé and Moragas, 1996; Kjellberg *et al*, 1997), with relatively low sensitivity rates ranging from 43 (Mårtensson *et al*, 1985) to 71% (Nance *et al*, 1991), averaging 58% (Motherby *et al*, 1999). Diagnostic inaccuracies and a high incidence of false negatives surely endanger patients with clinical mistreatment and mismanagement. Increasing the low sensitivity of PE cytology would be difficult to attain in the clinical area, as its diagnostic accuracy depends on the volume of liquid examined, the type of preparation and staining, the experience of the examiner and the number of sufficient specimens investigated. Rather, analyses that are both supplementary and complementary to cytology could be more clinically relevant resolutions for increasing the sensitivity rates of routine PE examinations for malignancy.

Accordingly, the investigation of tumour marker assays of sufficient clinical specificity and sensitivity for distinguishing between benign and malignant PEs emerged for improving the diagnostic value of cytology. A recent meta-analysis addressing the diagnostic accuracy of tumour markers for malignant PE found that the sensitivity and specificity of such markers were relatively low, ranging between 25 and 55%. Although these findings pave the way for enhancing the diagnostic value of cytology, the reported markers were unspecific to lung cancer, the main cause of MPE, enforcing the necessity for additional investigation of more sensitive biological markers (Liang *et al*, 2008).

The aim of this study was to investigate pleural fluid for various biological markers that would allow, once supplemented to the cytological analysis, a highly sensitive distinction between benign and malignant PEs. As non-small-cell lung cancer (NSCCA) is the most common cause of MPE (Neragi-Miandoab, 2006), and to minimise the variability of the study sample and increase its homogeneity, we compared a group of patients diagnosed with lung cancer with patients diagnosed with non-malignant inflammatory pathologies.

## Materials and methods

### Patients and study design

We analysed data from a study group of 19 patients diagnosed with lung cancer, and compared it with a group of 22 patients suffering from various benign inflammatory pathologies. The latter group included patients with inflammatory pathology of the pleura, secondary to trauma, pneumonia or TB. The lung cancer group included 12 men and 7 women with a mean age of 67.8±4.3 years and the non-malignant group included 15 men and 7 women with a mean age of 58.8±7.3 years (not significantly different). Among the lung cancer group were nine patients diagnosed with adeno-Ca, five with non-small-cell Ca (not specified), four with squamous-cell Ca and one with large-cell Ca. No cancer cells were found in the pleural fluid harvested from six patients, similar to all cases of the benign group (sensitivity and specificity rates of 68 and 100%, respectively). The definitive diagnosis of malignant diseases was verified in those six patients whose cytological analysis was negative by further tissue analysis procedures. Pleural fluid samples were collected and stored at −80°C until further use. The protein levels of matrix metalloproteinase (MMP)-9, MMP-3, MMP-2, p27, Skp2, Cox-2, CyclinD1 and Ki67, as well as the levels of carbonyls and the antioxidative capacity (IMANOX assay), were analysed. In addition to these 10 specific markers, presumably related to cancer/proliferation/oxidative stress, a routine analysis of the patient's blood and pleural fluid was performed. The routine pleural analysis included pH, albumin, LDH, glucose, total protein, amylase and white blood cells count (WBC). Albumin, total protein and WBC were also analysed in the patients’ blood. Interestingly, none of the routine parameters analysed were significantly different between the two groups (neither in the blood nor in the pleural fluid).

### Immunoreactivity assay

Pleural fluid samples were centrifuged (800 × g, 10 min, 4°C), and pellets were suspended in 150 *μ*l of lysis buffer (45 mM HEPES, 0.4 M KCl, 1 mM EDTA, 10% glycerol, pH 7.8). After 30 min of incubation at room temperature, the samples were centrifuged (11 000 × g, 10 min, 4°C). Protein concentrations in the supernatants were determined. A volume containing 50 ng of protein was transferred to a 1.5 ml vial and all samples were brought to the same volume of 500 *μ*l with the addition of PBS. The solutions were mixed well and 100 *μ*l of each sample was added to ELISA-plate wells (nunc-immunoplate; Thermo Fisher Scientific, Waltham, MA, USA). The plate was covered and stored overnight at 4°C. The next day, each well was washed three times with 100 *μ*l PBS-Tween solution (PBS-T, PBS containing 0.05% Tween 20) and a volume of 100 *μ*l of 1% BSA PBS-T blocking solution (PBS containing 0.05% Tween 20 and 1% BSA) was added to each well. After 1 h incubation at room temperature, 100 *μ*l of primary antibody was added to each well. After 2 h of incubation at room temperature, the plate was washed as described above and a volume of 100 *μ*l of secondary antibody was added to each well. After 2 h of incubation at room temperature, the plate was washed as described above. To achieve colour development, we added 100 *μ*l of 3,3′,5,5′-tetramethylbenzidine solution (TMB) (Southern Biotech, Birmingham, AL, USA) to each well. After 1–2 min, we added 100 *μ*l of stopping reagent to each well (10% sulphuric acid). Absorbencies of the samples, representing the levels of the specific proteins examined, were measured at 450 nm, directly after the addition of the stopping reagent, using a Zenith 200 ELISA reader (Anthos, Eugendorf, Austria). For MMP-9 we used a polyclonal rabbit anti-human antibody (1 : 1000; Sigma-Aldrich, Saint Louis, MO, USA); for MMP-3 we used a polyclonal rabbit anti-human antibody (1 : 500; Sigma-Aldrich); for MMP-2 we used a monoclonal rabbit anti-human antibody (1 : 1000; Sigma-Aldrich); for p27 we used a polyclonal rabbit anti-human antibody (1 : 1000; Cell Signaling, Danvers, MA, USA); for Cyclin D1 we used a polyclonal rabbit anti-human antibody (1 : 500; Sigma-Aldrich); for Ki67 we used a monoclonal rabbit anti-human antibody (1 : 1000; Acris Antibodies, Herford, Germany); for Cox2 we used a polyclonal rabbit anti-human antibody (1 : 1000; Cell Signaling); for Skp2 we used a polyclonal rabbit anti-human antibody (1 : 1000; Cell Signaling); and for all assays we used a peroxidase-conjugated goat anti-rabbit secondary antibody (1 : 5000; Jackson Immunoresearch, West Grove, PA, USA).

### Detection of protein oxidation (protein carbonyl assay)

An enzyme-linked immunosorbent assay (ELISA) colorimetric test kit (BioCell Corporation, Papatoetoe, New Zealand) was used to quantitatively measure the products of protein oxidation (carbonyls) in pleural fluid samples. Samples were centrifuged (800 × g, 10 min, 4°C) and pellets were suspended in 150 *μ*l of lysis buffer (45 mM HEPES, 0.4 M KCl, 1 mM EDTA, 10% glycerol, pH 7.8). After 30 min incubation at room temperature, the samples were centrifuged (11 000 × g, 10 min, 4°C) and supernatants were stored at −20°C. On the day of the carbonyl analysis, the supernatants were thawed and protein concentrations were determined. A volume of 20 *μ*g was transferred to a 1.5 ml vial and all samples were brought to the same volume of 100 *μ*l with the addition of water of high-pressure liquid chromatography grade. We added 0.8 volumes of ice-cold 28% trichloroacetic acid, mixed well, and after 10 min of incubation on ice , the tubes were centrifuged (10 000 × g, 3 min, 4°C). Supernatants were carefully aspirated without disturbing the pellet. A volume of 5 *μ*l of EIA buffer (1 M phosphate solution containing 1% BSA, 4 M NaCl, 10 mM EDTA and 0.1% sodium azide) and 15 *μ*l of diluted 2,4-dinitrophenol (DNP) solution were added to samples according to the manufacturer's instructions. After 45 min of incubation at room temperature, 5 *μ*l of each sample was taken to a parallel set of 1.5 ml vials containing 1 ml EIA buffer. The solutions were mixed well and 200 *μ*l of each sample was added to ELISA-plate wells. The plate was covered and stored overnight at 4°C. The next day, the plate was washed three times with EIA buffer (250 *μ*l per well) and 250 *μ*l of diluted blocking solution (provided by the manufacturer) was added to each well. After 30 min incubation at room temperature, the wells were washed as described above and 200 *μ*l of diluted anti-DNP-biotin antibody was added to each well. The plate was incubated for 1 h at 37°C. After incubation, the plate was washed and 200 *μ*l of diluted streptavidin-HRP was added to each well. After 1 h incubation at room temperature, the plate was washed as described above. To achieve colour development, we added 200 *μ*l of chromatin reagent (provided by the manufacturer) to each well. After 5 min, we added 100 *μ*l of stopping reagent to each well. The absorbencies of samples were measured at a wavelength of 450 nm directly after the addition of the stopping reagent, using a Zenith 200 ELISA reader (Anthos). To quantify absorbance values, we performed the same procedure for standard and control samples provided by the manufacturer, and created a standard curve.

### Antioxidative capacity (IMANOX assay)

An ELISA colorimetric test kit (Immundiagnostik AG, Bensheim, Germany) was used to quantitatively measure the total antioxidative capacity in pleural fluid samples, as previously described (Hershkovich *et al*, 2007). Briefly, a defined amount of exogenous hydrogen peroxide (H_2_O_2_) was added to the samples, which was partly eliminated by the pleural fluid antioxidants. The residual H_2_O_2_ was determined by an enzymatic reaction involving the conversion of TMB to a colorimetric product. After the addition of a stop solution to the samples, absorbance was measured at 450 nm, using a Zenith 200 ELISA reader (Anthos). To quantify the absorbance values, we used a calibrator provided by the manufacturer.

### Statistical analysis

The levels of various markers were evaluated in pleural fluid samples, and mean s.d. (STD and mean standard error (s.e.) values were analysed and compared with the two sample *t*-tests for differences in means. The criterion for statistical significance was *P*<0.05. The correlations between the levels of various markers in the pleural fluid samples were analysed using the Pearson correlation analysis. A correlation matrix of estimators was used to analyse the correlation coefficients between parameters. For classification analysis, cutoff values were calculated as mean±1 s.d. of the non-malignant patients. Sensitivity and specificity values were calculated as the fraction of observations that was correctly classified.

## Results

### Pleural fluid markers

**Figure 1:**
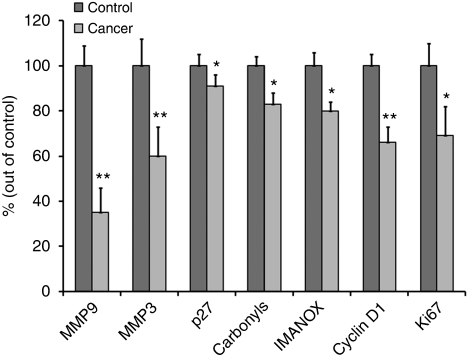
Mean+s.e. levels of the seven significantly altered parameters in the pleural fluid of lung cancer patients (cancer) compared with benign inflammatory patients (control), presented as a percentage of controls (^*^P<0.05; ^**^P<0.01).

Pleural fluid marker analysis revealed highly significant differences in the levels of seven of the analysed markers between benign and lung cancer patients (Table 1, Figure 1): the concentrations of all seven markers, MMP-9, MMP-3, CycD1, Ki67, ImAnOx, carbonyls and p27, were lower in cancer patients by 9–65%. In benign patients, the pleural fluid mean (±s.e.) concentrations (OD values) of MMP-9, MMP-3, CycD1, Ki67, ImAnOx, carbonyls and p27 were 0.426±0.037, 0.202±0.024, 0.799±0.041, 0.294±0.031, 205.9±12.3, 0.802±0.032 and 0.597±0.031, respectively. The decreases in pleural MMP-9, MMP-3 and CycD1 were profound and highly significant, by 65%, *P*=0.000003; by 40%, *P*=0.0007; and by 34%, *P*=0.0001. The mean concentration of Ki67 was lower in the PE of cancer patients by 31% (*P*=0.028) and that of ImAnOx, carbonyls and p27 was lower by 20% (*P*=0.012), 17% (*P*=0.025) and by 9% (*P*=0.025), respectively (Table 1). Interestingly the pleural concentrations of the other three markers analysed were also lower in the lung cancer group than in the benign group, yet not in a statistically significant manner. Thus, the concentrations of MMP-2, Skp-2 and Cox-2 were reduced by 23% (*P*=0.238), 8% (*P*=0.160) and 10% (*P*=0.132), respectively.

The sensitivity values of these seven analysed markers were in the range of 33–100%, whereas the specificity values were in the range of 77–90% (Table 1).

Sensitivity and specificity values were especially high for MMP-9 (100 and 80%, respectively) and CycD1 (82 and 87%, respectively). High specificity values were also found for IMANOX and MMP-3 (90 and 87%, respectively). The positive likelihood ratio (LR+), the negative likelihood ratio (LR−) and the diagnostic odds ratio values of MMP-3 and of IMANOX were 6.9, 0.1 and 62.7, respectively. These values for CycD1 were 6.3, 0.2 and 31.5, respectively. The LR+ and LR− values of MMP-9 were 5 and 0, respectively.

Multiple significant (<−0.4 or *r*>0.4) correlations were demonstrated among all seven markers, each with some of the others. The most significant correlations were demonstrated between MMP-9 and CycD1 (0.69); MMP-9 and MMP-3 (0.68); and MMP-9 and Ki67 (0.62). Also quite high were the significant correlations between Ki67 and p27 (0.57) and between Ki67 and carbonyls (0.52) (Table 2).

### Cytological and biochemical examination

The 19 cancer patients included the following three subgroups: nine patients with a definitive diagnosis of NSCCA/Adeno Ca/SCC; four patients with a non-definitive malignant diagnosis or a suspected malignant diagnosis; and six patients with a benign cytological diagnosis identical to that obtained in the 22 benign patients. In all 19 cancer patients, the final diagnosis was made on tissue analysis obtained in a biopsy or in a fine-needle aspiration procedure. The non-malignant cells observed in these six false negatives and in the 22 truly benign cases included mesothelial cells, lymphocytes, leukocytes, granulocytes and histiocytes.

The pleural biochemical analysis revealed the following mean±STD values in the control group: Ph =7.4±0.2, albumin=2±0.9 (mg l^−1^), LDH=172±61 (IU l^−1^), glucose=129±67 (mg per 100 ml), total protein=3.8±1.4 (mg per 100 ml) and amylase=30.4±16.9 (10^−2^ IU l^−1^). WBC was 5.6±3.2. As previously mentioned, these values were not statistically different from those found in cancer patients.

## Discussion

In this study, we examined lung NSCCA patients as they comprise the largest lung cancer group (85% of all cases), owing to the fact that lung cancer is the most common cause of MPE (Neragi-Miandoab, 2006). Furthermore, investigation of patients with a common pathology contributed to the homogeneity of the study sample, as accurate and early diagnosis of their staging may promote effective clinical management. Such a diagnosis based on a simple PE examination may be achieved rapidly and may substantially reduce the rate of accompanying morbidity.

The most important result in this study involves the pleural fluid concentrations of seven analysed biological markers, found to be significantly lower in lung cancer patients than in benign inflammatory patients. This was especially true for MMP-9, MMP-3 and CycD1, the concentrations of which in cancer patients were lower by 65 (*P*<0.000003), 40 (*P*<0.0007) and 34% (*P*<0.0001), respectively. High rates of sensitivity and specificity were found for these markers, which for MMP-9, MMP-3 and CycD1 were 80 and 100% 87 and 73% and 87 and 82%, respectively. We thus believe that the demonstrated results are of significant merit with respect to both the clinical and pathogenesis-related aspects of lung cancer.

As for the clinical aspect, the significant differences demonstrated in pleural markers of lung cancer and benign patients may be used as a diagnostic tool, which is of special importance for patient staging and management. Accordingly, PE analysis may become a valuable rapid-diagnosis tool, saving many unnecessary biopsies and hospital/outpatient clinic visits, especially in light of the poor sensitivity of the currently available pleural cytological examination. It is well established that the merit of any compositional analysis is further augmented when a concurrent analysis is performed simultaneously for several markers, thus reaching higher sensitivity and specificity rates (Shpitzer *et al*, 2009). For example, in this study, the specificity rate of the PE analysis reached 100% when the analysis included all markers simultaneously, rather than one.

Along this line of thought, a patient with a negative cytological finding and a pleural-marker profile supporting benign disease would receive a different treatment than a patient with a negative cytological finding but with a pleural-marker analysis supporting lung cancer. In the latter case, the clinician will have a higher index of suspicion and would have sufficient grounds for requesting a repeat cytological analysis to reassess and consequently confirm the patient's clinical condition.

As for the pathogenesis-related merit of these findings, it is noteworthy that never before had the currently reported markers been concomitantly analysed in pleural fluid and most of them had never been examined in PE at all. To our knowledge, only MMP-2 and MMP-9 were previously analysed in the pleural fluid of lung cancer and benign inflammatory patients (Kotyza *et al*, 2004; Di Carlo *et al*, 2005, 2007; Iglesias *et al*, 2005; Park *et al*, 2005; Vatansever *et al*, 2009). We decided to analyse these markers as they belong to three different marker groups that may be related to cancer and/or to inflammatory diseases. p27, Skp2, Cox2, CycD1 and Ki67 are considered as tumour and/or proliferation markers (Huang *et al*, 2003; Siggelkow *et al*, 2004; Adjei, 2005; Vig-Varga *et al*, 2006; Garg *et al*, 2008; Park *et al*, 2008; Chang *et al*, 2009; Meeran *et al*, 2009; Mukhopadhyay *et al*, 2009), ImAnOx and carbonyls are oxidative markers (Nyström, 2005; Bahar *et al*, 2007) and MMP-9, -3 and -2 are MMPs that are expressed in various pathological processes such as inflammation and cancer and also in response to infections (Korpos *et al*, 2009; Oikonomidi *et al*, 2009; Vanlaere and Libert, 2009). Enhanced oxidative stress characterises both inflammation and cancer; all other markers analysed were also shown previously to be associated with inflammation, as well as with oxidative stress, presumably mediated through the activation of NF-*κ*B (Huang *et al*, 2003; Vig-Varga *et al*, 2006; Park *et al*, 2008; Gonda *et al*, 2009; Karalis *et al*, 2009; Ku *et al*, 2009; Perricone *et al*, 2009; Víctor *et al*, 2009). Interestingly, the significantly altered markers correlated among themselves (Table 2), indicating that they were all associated with each other, prompting hypothesis that they might belong to a single biological network that, when fully understood, may be used for the development of drugs related to lung cancer. Alternatively, they may all share another feature that is not related to a single biological pathway but rather to a single general condition, affecting the cancer *vs* benign groups in a different manner. Such a condition may be related to the time period of the existing pathology, that is, its chronicity, or to the volume of the exudates effused. Hence, if the volume of the PE medium (transudate transfused) analysed is larger in cancer patients, one would expect all the markers analysed to be significantly diluted and for their concentrations to be significantly reduced. Support for this suggestion may be found in the fact that all 10 different markers analysed were lower in the cancer group, although this difference did not always reach statistical significance.

Regardless of the mechanism involved, the clinical significance of the presented results is substantial. Being able to significantly improve the rapidity and sensitivity of the PE examination for diagnosis and staging of NSCCA patients is of paramount importance. In spite of the relatively high statistically significant *P-*values obtained, further research analysing a larger group of patients is warranted, as the number of patients currently examined is rather limited. Further research aimed at finding other sensitive biological markers in PE and for defining the best combination for marker analysis is warranted as well. The relative ease in performing the analysis of these markers in any hospital laboratory may turn such an analysis into a popular new diagnostic tool in the clinical setup.

## Figures and Tables

**Table 1:** Statistical analysis of seven analysed parameters

**Parameter**	**% Of change (out of benign patients)**	***P***	**Specificity (%)**	**Sensitivity (%)**
MMP 9	−65	0.000003	80	100
MMP 3	−40	0.0007	87	73
p27	−9	0.025	80	33
Carbonyls	−17	0.025	80	38
IMANOX	−20	0.012	90	58
Cyclin D1	−34	0.0001	87	82
Ki67	−31	0.028	77	50

Abbreviations: MMP 9=matrix metalloproteinase-9; MMP 3=matrix metalloproteinase-3; IMANOX=antioxidative capacity.

The concentrations of the seven parameters that were found to be significantly lower in the pleural fluid of lung cancer patients as compared with non-malignant patients. The following were calculated: percentage of change in the mean levels of each parameter, statistical significance of the change (represented by *P*), the specificity and sensitivity of each parameter.

**Table 2:** Correlation rates (moderate–high) between the significantly altered various parameters

**Parameters compared**	***r***
MMP-9–MMP 3	0.68
MMP-9–cyclin D1	0.69
MMP-9–Ki67	0.62
MMP-3–IMANOX	0.41
MMP3–cyclin D1	0.48
Ki67–p27	0.57
Ki67–carbonyls	0.52

Abbreviations: *r*=Pearson correlation coefficient; *r*<−0.4 or *r*>0.4=significant correlation; MMP-9=matrix metalloproteinase-9; MMP-3=matrix metalloproteinase-3; IMANOX=antioxidative capacity.
